# Improving the evidence for indicator condition guided HIV testing in Europe: Results from the HIDES II Study – 2012 – 2015

**DOI:** 10.1371/journal.pone.0220108

**Published:** 2019-08-13

**Authors:** Dorthe Raben, Ann Kathleen Sullivan, Amanda Mocroft, Galyna Kutsyna, Vesna Hadžiosmanović, Anna Vassilenko, Nikoloz Chkhartisvili, Viktar Mitsura, Court Pedersen, Jane Anderson, Josip Begovac, Ulrik Bak Dragsted, Barbara Bertisch, Anna Grzeszczuk, Jane Minton, Valentina Coca Necsoi, Maria Kitchen, Faiza Ajana, Anton Sokhan, Laura Comi, Paymaneh Farazmand, Dragica Pesut, Stephane De Wit, José Maria Gatell, Brian Gazzard, Antonella d’Arminio Monforte, Jürgen Kurt Rockstroh, Yazdan Yazdanpanah, Karen Champenois, Marie Louise Jakobsen, Jens Dilling Lundgren, on behalf of the HIDES Study Group

**Affiliations:** 1 Centre for Health & Infectious Disease Research, Rigshospitalet, Copenhagen, Denmark; 2 Chelsea and Westminster Hospital, NHS Foundation Trust, London, England, United Kingdom; 3 University College London, London, England, United Kingdom; 4 Luhansk AIDS Center, Luhansk, Ukraine; 5 Clinical Center University of Sarajevo, Infectious Diseases Clinic, Sarajevo, Bosnia; 6 Belarusian State Medical University, Minsk, Belarus; 7 AIDS & Clinical Immunology Research Center, Tiblisi, Georgia; 8 Gomel State Medical University, Gomel, Belarus; 9 Odense University Hospital, Odense, Denmark; 10 Homerton University Hospital, London, England, United Kingdom; 11 University Hospital of Infectious Diseases, Zagreb, Croatia; 12 Zealand University Hospital, Roskilde, Region Zealand, Denmark; 13 Kantonsspital, St. Gallen, Switzerland; 14 Medical University of Bialystok, Bialystok, Poland; 15 St James’s University Hospital, Leeds, England, United Kingdom; 16 Saint-Pierre University Hospital, Université Libre de Bruxelles, Brussels, Belgium; 17 University Hospital Innsbruck, Innsbruck, Austria; 18 Centre Hospitalier de Tourcoing, Tourcoing, France; 19 Kharkiv National Medical University, Kharkiv, Ukraine; 20 Ospedale di Bergamo, ASST Papa Giovanni XXIII, Bergamo, Italy; 21 Huddersfield Royal Infirmary, Huddersfield, England, United Kingdom; 22 University of Belgrade School of Medicine, Clinical Centre of Serbia, Belgrade, Serbia; 23 Hospital Clinic de Barcelona/IDIBAPS, University of Barcelona, Barcelona, Spain; 24 Unit of Infectious Diseases, San Paolo Hospital, Milan, Italy; 25 HIV Outpatient Clinic, University of Bonn, Bonn, Germany; 26 Université Paris Diderot, Sorbonne Paris Cité, Paris, France; 27 Hôpital Bichat, Paris, France; Boston University, UNITED STATES

## Abstract

**Background:**

It is cost-effective to perform an HIV test in people with specific indicator conditions (IC) with an undiagnosed HIV prevalence of at least 0.1%. Our aim was to determine the HIV prevalence for 14 different conditions across 20 European countries.

**Methods:**

Individuals aged 18–65 years presenting for care with one of 14 ICs between January 2012 and June 2014 were included and routinely offered an HIV test. Logistic regression assessed factors associated with testing HIV positive. Patients presenting with infectious mononucleosis-like syndrome (IMS) were recruited up until September 2015.

**Results:**

Of 10,877 patients presenting with an IC and included in the analysis, 303 tested positive (2.8%; 95% CI 2.5–3.1%). People presenting with an IC in Southern and Eastern Europe were more likely to test HIV positive as were people presenting with IMS, lymphadenopathy and leukocytopenia/ thrombocytopenia. One third of people diagnosed with HIV after presenting with IMS reported a negative HIV test in the preceding 12 months. Of patients newly diagnosed with HIV where data was available, 92.6% were promptly linked to care; of these 10.4% were reported lost to follow up or dead 12 months after diagnosis.

**Conclusion:**

The study showed that 10 conditions had HIV prevalences > 0.1%. These 10 ICs should be adopted into HIV testing and IC specialty guidelines. As IMS presentation can mimic acute HIV sero-conversion and has the highest positivity rate, this IC in particular affords opportunities for earlier diagnosis and public health benefit.

## Introduction

The HIDES 1 study (HIV Indicator Diseases across Europe Study) gave proof of concept that Indicator Condition (IC) guided HIV testing is an acceptable, feasible and effective strategy to diagnose people living with HIV in health care setting encounters [1]. Identification of ICs that should prompt a routine offer of an HIV test in all health care settings is based on their classification into three categories; conditions which are AIDS defining among people living with HIV (Category 1); conditions with a proven HIV prevalence ≥ 0.1% (Category 2a) or expected to do so (Category 2b) and conditions where not identifying HIV infection can have significant adverse implications for the management of the patient (e.g. chemotherapy; Category 3). The 0.1% threshold for Category 2 is based on studies suggesting that HIV testing is cost-effective when the undiagnosed HIV prevalence in the tested population is at least 0.1% [2–3]. The implementation of this strategy is further described in the Guidance for Implementing HIV Testing in Adults in Health Care Settings developed for the European Region [4–5]. The pilot study HIDES 1, which informed the guidance evidence base, demonstrated eight of the ICs studied fulfilled the study’s criteria having an HIV prevalence of ≥ 0.1%, i.e. sexually transmitted infections (STI), malignant lymphoma, irrespective of type (LYM), cervical or anal cancer/dysplasia (CAN), hepatitis B or C virus infection (HEP), herpes zoster (HZV), ongoing infectious mononucleosis-like syndrome (IMS), unexplained leukocytopenia/thrombocytopenia lasting >4 weeks (CYT), seborrheic dermatitis/exanthema (SEB). An audit of HIV testing in tuberculosis (TB), candida oesophagitis (OC), non-Hodgkin’s lymphoma (NHL), anal cancer (AC) cervical cancer (CC) and hepatitis B and C (HEP) across Europe was conducted in parallel and is reported elsewhere [6]; this demonstrated poor performance of IC guided testing in people presenting with well recognised AIDS-defining and non-AIDS defining indicator conditions.

The aim of this second phase of the study, HIDES II, was to expand the testing strategy by increasing the number of both ICs and participating centres to determine the HIV prevalence for 14 different conditions across Europe and thereby further refining the evidence for Category 2 ICs. Secondary outcomes included CD4 count and disease stage at diagnosis and access to care for those individuals testing HIV positive.

## Methods

Thirteen ICs were selected for determination of undiagnosed HIV prevalence. Individuals aged 18–65 years, not known to be HIV positive and presenting for care with one of the 14 ICs listed below between January 2012 and June 2014 were included:

- malignant lymphoma (irrespective of type)
- cervical dysplasia or cancer (CIN II and above)
- anal dysplasia or cancer
- hepatitis B infection (HBV, acute or chronic; irrespective of when diagnosed)
- hepatitis C infection (HCV, acute or chronic; irrespective of when diagnosed)
- both hepatitis B and C infection (acute or chronic; irrespective of when diagnosed)
- infectious mononucleosis-like syndrome (IMS)
- unexplained leukocytopenia or thrombocytopenia of at least 4 weeks duration
- seborrheic dermatitis/exanthema
- pneumonia, requiring hospital admission for at least 24 hours
- unexplained lymphadenopathy
- peripheral neuropathy of unknown cause (diagnosed by neurologist)
- primary lung cancer and
- severe or recalcitrant psoriasis (newly diagnosed).

Given the importance of infectious mononucleosis-like syndrome (IMS) as an IC in relation to recency of infection, transmission risk and high HIV prevalence the IMS arm was extended until September 2015.

Recruitment was continued at sites involved in HIDES 1 [1], with additional sites recruited by invitation through established contacts (full list of sites and settings in S1 Appendix). Ethical approval was obtained where required in participating countries in line with National requirements. As HIV testing was performed routinely as part of the investigation of the IC, consent to the test was obtained verbally where required (list of EC/IRB per country in S2 Appendix). Consecutive patients were enrolled if they presented with the selected IC and were not already known to be HIV positive. Patient inclusion was based on the treating physician’s clinical, microbiological or histological diagnosis, or when the IC was part of the differential diagnosis. Data were captured through the electronic submission system REDCap (Vanderbilt University, Nashville, USA) (HIDES patient enrolment form in S3 Appendix).

Individual’s characteristics were compared across regions using chi-squared tests for categorical variables and the Wilcoxon test for continuous variables. Clinics were grouped into four regions, consistent with those used in the EuroSIDA study [7]; Southern Europe (Greece, Italy, Spain, Israel), Central Europe (Belgium, France, Germany, Switzerland, Austria), Northern Europe (UK, Denmark, Netherlands) and Eastern Europe (Georgia, Belarus, Serbia, Poland, Romania, Ukraine, Croatia, Bosnia and Herzegovina). The prevalence of testing HIV positive was calculated, and 95% and 99% confidence intervals are shown. Logistic regression was used to determine factors associated with testing HIV positive. Due to small numbers testing or testing HIV-positive, we combined malignant lymphoma, anal dysplasia/cancer; hepatitis B+C, seborrheic dermatitis/exanthema, peripheral neuropathy of unknown cause, primary lung cancer and severe or recalcitrant psoriasis into one ‘other’ category and used pneumonia as the reference category as the largest group (excluding the IMS extension). In sensitivity analyses, the prevalence of testing HIV-positive was assessed separately in the IMS extension, and after excluding data from the three largest sites.

A priori, the interaction between region and indicator conditions was of interest and formally tested. In this analysis, due to the low numbers of individuals from Southern, Central and Northern Europe, these regions were combined and compared to Eastern Europe, and only ICs with more than 30 HIV positive results were considered (pneumonia, IMS and hepatitis B and C combined where those testing for hepatitis B alone, hepatitis C alone and both hepatitis B and C were combined into a single group).

Logistic regression was also used to determine the factors associated with late presentation for HIV, defined as a CD4 count ≤ 350 cells/mm^3^ at the time of HIV diagnosis [8]. Forward selection was used for this latter model, with p<0.1 as the entry criteria, with the exception of including region and indicator conditions as variables of interest. In both logistic regression analyses, the largest categories were chosen as the reference groups and some categories were combined due to small numbers of individuals testing HIV positive. All ICs with fewer than 10 individuals testing HIV positive were combined to give the ‘other’ category.

Data on CD4 count, disease stage and linkage to care were collected for those with a confirmed HIV diagnosis where available. Not all centres were able to retrieve this information as, on receipt of a positive test result, many individuals transferred their care to an HIV treatment service. This separation of services presented barriers to obtaining subsequent clinical information in some instances. We have analysed linkage to care for individuals with data available and again treating missing data as failure of linkage to care. Linkage to care was defined as a patient being seen for specialist HIV care after diagnosis with date of first CD4 count being a proxy for first clinic visit, while treatment initiation was defined as the date of starting antiretrovirals as reported by clinic. Time taken for linkage to occur (measured as time between date of HIV diagnosis and date of first CD4 count) was considered prompt when this was less than three months (<91 days) [9]. As many of the sites recruiting people into the study were non-traditional HIV testing sites, the feasibility and clinical safety of both testing and linkage to care was important to investigate in order to address concerns regarding reduced or delayed access to treatment and care.

All analyses were performed using SAS (Statistical Analysis Software version 9.4).

## Results

### Patient characteristics

Of 11,663 potentially eligible individuals, 10,877 were included in the analysis (93.3%); of the 786 who were excluded, 671 were outside the age range, 68 had no IC information, 30 had no HIV test result, 11 were missing the date of their HIV test and 6 had missing gender. The IMS extension accounted for 1,111 (10.2%) eligible individuals.

Table 1 shows the participant’s characteristics overall and stratified by region; of the 10,877 individuals, 576 (5.3%) were from Southern Europe and 1,006 (9.2%), 2,822 (25.9%) and 6,473 (59.5%) were from Central, Northern and Eastern Europe respectively. There was considerable heterogeneity between regions; Eastern Europe participants were more likely to be younger, white Caucasians, less likely to have ever tested for HIV and more likely to have tested in an in-patient setting (p<0.0001).

**Table 1:** Characteristics of included participants stratified by region.

		All		South		Central		North		East		P
		N	%	N	%	N	%	N	%	N	%	
All		1087710877	100100	576576	5.35.3	10061006	9.29.2	28222822	25.925.9	64736473	59.559.5	
Gender	Male	58595859	53.953.9	311311	54.054.0	592592	58.858.8	14091409	49.950.1	35473547	54.845.2	<0.0001
	Female	50185018	46.146.1	265265	46.046.0	414414	41.241.2	14131413	50.162.8	29262926	45.254.8	
Ethnicity	Caucasian	93679367	86.186.1	481481	83.583.5	682682	67.867.8	17721772	62.811.7	64326432	99.40.2	<0.0001
	Asian	376376	3.53.5	1212	2.12.1	1919	1.91.9	329329	11.78.7	1616	0.20.0	
	African	344344	3.23.2	77	1.21.2	9191	9.09.0	245245	8.716.9	11	0.00.4	
	Unknown	790790	7.37.3	7676	13.213.2	214214	21.321.3	476476	16.920.4	2424	0.410.1	
Setting	Outpatient	48634863	44.744.7	244244	42.442.4	475475	47.247.2	18891889	66.916.5	22552255	34.850.0	<0.0001
	In patient	41814181	38.438.4	244244	42.442.4	235235	23.423.4	465465	16.516.3	32373237	50.00.2	
	Prim. care	688688	6.36.3	7272	12.512.5	147147	14.614.6	459459	16.30.3	1010	0.215.0	
	Unknown	11451145	10.510.5	1616	2.82.8	149149	14.814.8	99	0.30.0	971971	15.00.0	
		Median	IQR	Median	IQR	Median	IQR	Median	IQR	Median	IQR	
Age	Years	36	26–48	39	30–51	41	31–52	38	29–51	33	26–44	<0.0001
Date	Mm/yy	7/13	1/13–2/14	6/13	11/12–2/14	8/13	12/12–1/14	9/13	3/13–4/14	6/13	1/13–1/14	<0.0001

Age calculated at date of blood sample date. IQR; interquartile range. P-value from chi-squared test for categorical variables and Wilcoxon test for continuous variables. Prim. Care; primary care. Mm/yy; calendar month and year.

### HIV prevalence

**Fig 1:**
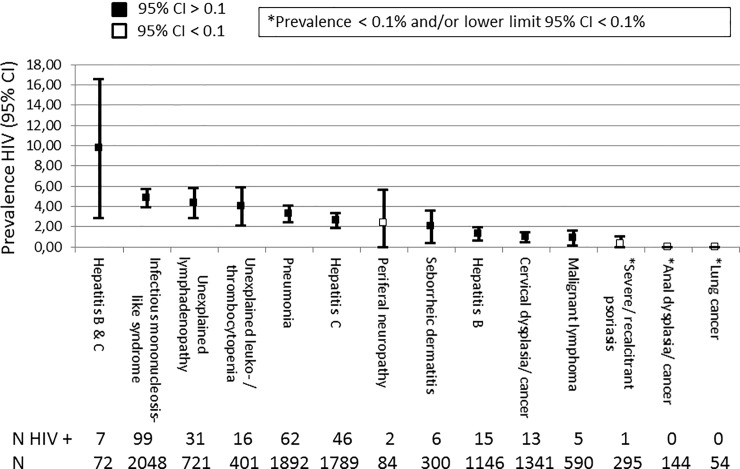
Prevalence of testing HIV positive: HIDES II study.

In total, 303 individuals tested HIV positive (2.8%; 95% confidence interval [CI] 2.5–3.1%). The proportion testing positive varied between regions, it was greater in Southern Europe (n = 27, 4.7%; 95% CI 3.0–6.4%), followed by Eastern Europe (n = 223, 3.4%; 95% CI 3.0–3.9%), Northern Europe (n = 41, 1.5%; 95% CI 1.0–1.9%) and lowest in Central Europe (n = 12, 1.2%, 95% CI 0.5–1.9%). The rates for different ICs are shown in Fig 1 and was highest for hepatitis B and C co-infection (9.7%; 95% CI 2.9–16.6%), albeit with wide confidence intervals reflecting the comparatively small size of this group, followed by IMS (4.8%; 95% CI 3.9–5.8%). Two ICs had no new HIV diagnoses; anal dysplasia/cancer and primary lung cancer. The lower limit of the 95% confidence interval exceeded 0.1% for all ICs, except for anal dysplasia/cancer, primary lung cancer, peripheral neuropathy and psoriasis. Two additional ICs, malignant lymphoma and seborrheic dermatitis/exanthema had lower 99% confidence limits below 0.1%.

**Fig 2:**
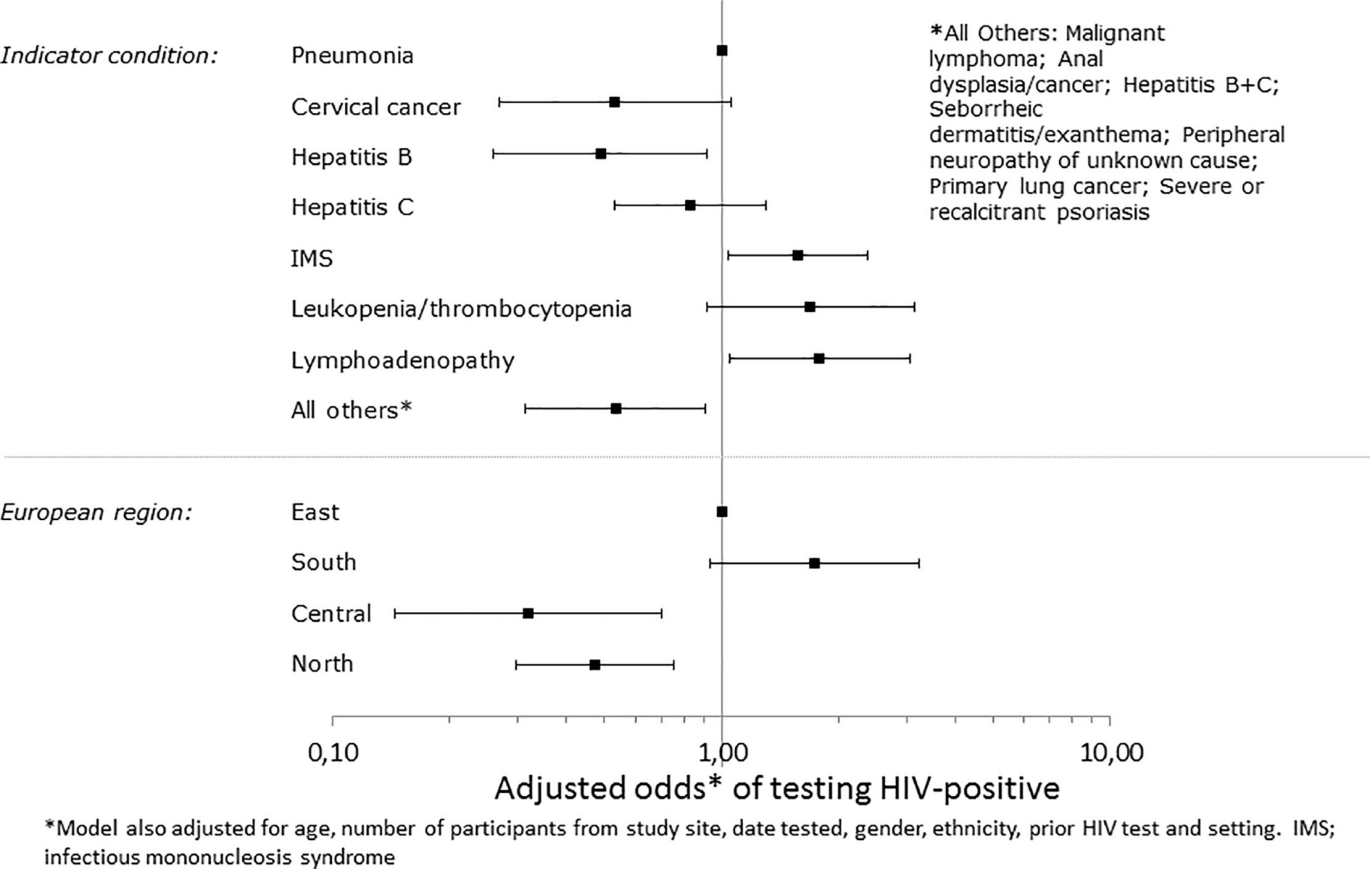
Adjusted odds of testing HIV positive.

Fig 2 shows the association between IC’s, region and testing HIV positive after adjustment for other factors. Compared to Eastern Europe, individuals from both Central and Northern Europe had lower odds of testing positive, while those from Southern Europe were more likely to test positive, although this was marginally statistically significant. Compared to pneumonia, those presenting with IMS, lymphadenopathy and leukocytopenia/ thrombocytopenia were more likely to test HIV positive, although this was marginally statistically significant for leukocytopenia/ thrombocytopenia, while those presenting with cervical cancer, hepatitis B alone, and the combined group of ‘Other IC’ (malignant lymphoma, anal dysplasia/cancer, both hepatitis B and C (acute and chronic), seborrheic dermatitis/ exanthema, peripheral neuropathy, primary lung cancer and psoriasis) were less likely to test HIV-positive, although this was marginally statistically significant for cervical cancer.

In a pre-planned analysis, we found differences between regions (East *vs* South, Central and North combined) and the odds of testing HIV-positive varied according to the three most common ICs (global p<0.0001, test for interaction). We therefore repeated the analysis stratified by IC’s. For pneumonia and hepatitis B and C combined, individuals from Eastern Europe were more likely to test HIV-positive compared to the other regions (adjusted odds ratio [aOR] 2.06; 95% CI 0.87–4.86 and 1.56; 95% CI 0.69–3.52 respectively). The opposite was seen for IMS, where individuals from Western Europe were less likely to test positive compared to other regions combined (aOR 0.87; 95% CI 0.40–1.88). These regional variations may reflect differences in the structure of the local health system, IC management and health seeking behaviours in addition to any true variation.

### Characteristics of those testing HIV positive and potential missed opportunities for earlier HIV testing

The median CD4 count at diagnosis, available for 268/303 (88.4%) was 230 cells/mm^3^ (IQR 95–430 cells/mm^3^) with no significant differences between regions (p = 0.15, table in S1 Table); the highest median CD4 count at diagnosis was seen in Northern Europe and lowest in Southern Europe. Of these 178 (66.4%) would be classified as late presenters based on CD4 count alone, and was highest in Eastern Europe, followed by South, Central, and Northern Europe respectively (p = 0.019). The median viral load (VL) at diagnosis (available for 265 individuals; 87.5%), was 5.2 log_10_copies/ml (IQR 4.6–5.8 log_10_copies/ml) with no significant differences between regions (p = 0.22) or when comparing the proportion with a high viral load (VL ≥100,000 copies/ml, p = 0.60).

Of the 293 individuals who provided information on previous HIV-associated symptoms 193 (65.9%) reported no symptoms (10 had missing data). Minor symptoms reported included infectious mononucleosis-like syndrome in 15, oral candidiasis in 35, herpes zoster in 15, unexplained leukocytopenia/ thrombocytopenia in 17 and seborrheic dermatitis in 15. Of the 223/294 individuals who provided information on previous STIs, 223 (75.9%) reported no prior STI in the past 5 years. Reported STIs included gonorrhoea (12), syphilis (11), other ulcerative genital conditions (12), chlamydia (11) and unspecified (36). Of the 280 individuals with available data on hospital admissions in the 5 years prior to their HIV diagnosis, 53 (18.9%) had been hospitalised. Twenty-five (47%) of these admissions were associated with an AIDS defining illness: herpes (chronic/extra-genital (5), recurrent bacterial pneumonia (4), oesophageal candidiasis (4), pulmonary tuberculosis (3), pneumocystis pneumonia (3), and one each of progressive multifocal leukoencephalopathy, non-Hodgkin’s lymphoma, recurrent salmonella, toxoplasmosis, histoplasmosis and cervical cancer. Twenty-three of those admitted reported other serious diagnoses, including pneumonia (13), endocarditis (3), bacteraemia (2), cervical dysplasia (2) and one each of lung, anal and breast cancer.

A total of 166 (54.8%) of those testing HIV positive reported a previous hospital admission for an AIDS defining illness, other serious illness, testing for HBV or HCV, or a diagnosed STI, representing opportunities for earlier testing and potential diagnosis of HIV.

### Infectious mononucleosis-like syndrome

The prevalence of HIV infection among those testing for IMS was consistently high across all regions; being highest in Southern Europe (9/89, 10.1%; 95% CI 1.7–18.5), followed by Eastern Europe (66/1169; 5.6%, 95% CI 4.3–7.0), Central Europe (2/61; 3.3%, 95% CI 0–7.7) and lowest in Northern Europe (22/729; 3.0%; 95% CI 1.8–4.3), with comparatively few individuals included from Central Europe.

Among those testing HIV positive, there were 61 individuals who reported a previous HIV test; of these, 11 (18.0%) were tested in 12 months preceding the positive test result. The proportion of those reporting a negative HIV test in the previous 12 months was greatest for IMS (10/31 32.3%), with only one person from all other ICs combined reporting a negative HIV test in the previous 12 months (1/30 3.3%), p = 0.00057.

### Linkage to care

Data on linkage to care and treatment initiation were reported for 176/303 (58.1%) individuals who tested HIV positive. Applying our definition, of those with available data, 163/176 (92.6%) were classified as being promptly linked to care and 6 (3.4%) as experiencing delayed linkage to care; for 7 there was no information available. Treating missing data as failure to link to care decreases the proportion successfully transferred to care to 53.8%.

Four (2.5%) of those promptly linked to care were subsequently recorded as lost to follow-up (LTFU) and 13 (8.0%) were recorded as having died within 12 months of diagnosis, giving a total of 17/163 (10.4%) of individuals with prompt linkage to care who were LTFU or dead within 12 months after diagnosis. Of these 17, 15 had information on CD4 count and 14 (93.3%) were diagnosed at a late stage; 3 of the 4 LTFU (95.0%) and 11 of those who died (100.0%).

### Sensitivity analysis

The analyses were repeated excluding the three largest sites (one each from Ukraine, Bosnia and Herzegovina, and the UK) to determine the extent to which data from the largest centres were explaining our findings. This analysis included 6,187 individuals (56.9%); of whom 159 tested HIV positive (rate 2.6%; 95% CI 2.2–3.0%). In a separate analysis, excluding just the IMS extension limited the analysis to 9,766 persons, produced an HIV prevalence of 2.6% (95% CI 2.3–3.0%), and a prevalence rate for IMS of 5.8% (54/937; 95% CI 4.3–7.3%). Results from both sensitivity analyses were very similar to our overall findings.

## Discussion

### HIV prevalence and regional differences

The HIDES study confirms a greater than 0.1% HIV prevalence in people presenting with certain ICs and confirms the feasibility of this testing strategy across Europe [1, 10–11]. The Guidance document on implementing IC guided HIV testing in healthcare settings in Europe [4] has three categories of ICs, where Category 2 is divided between those conditions associated with a confirmed undiagnosed HIV prevalence of >0.1% and thus testing is strongly recommended (2a), and those conditions considered likely to have this prevalence, but without proven evidence and hence testing should be offered (2b). Based on the results of this study, two more conditions; unexplained lymphadenopathy and community acquired pneumonia should be moved from the offer of a test to strongly recommending testing. The dissemination of the updated table of ICs is important and for highest impact could be specifically directed towards specialties involved in the treatment of these particular conditions, for example respiratory/pulmonology, haematology and internal medicine, as well as general practitioners.

Most HIV testing guidelines already recommend routine testing for specific at-risk populations, for example people who inject drugs (PWID), men who have sex with men (MSM), some ethnic groups; IC guided HIV testing should be incorporated as an additional part of a national strategy to improve testing offer in health care facilities. This approach has the benefit of being opportunistic; relying on the attendance of the individual for another health-related concern and hence does not require self-identification of risk or interventions by the health service to ‘reach’ them. It further normalises HIV testing with routine offer to all those presenting with the IC and is therefore not based on personal characteristics or behaviours, which can be both personally stigmatising and present barriers to health care professionals.

The proportion of individuals testing HIV-positive varied between regions, being higher in Southern and Eastern Europe and lower in Northern and Central regions. These differences are likely to be due to a combination of higher underlying HIV prevalence, higher levels of late diagnosis, differences in health care systems and settings were testing was performed and the ICs targeted in the respective regions.

### Late diagnosis and missed opportunities for earlier HIV testing

The proportion of individuals diagnosed HIV positive with a CD4 count less than 350 cells/uL was less than 50% only for IMS. In all other conditions the proportion of late presenters was greater than 75%, reflecting the opportunity IMS affords for earlier diagnosis with less potential for immunological damage.

The study observed a consistently high HIV prevalence for people presenting with IMS and considerably higher than the 0.1% cost-effectiveness cut-off, highlighting that routine HIV testing in this group represents a very effective HIV case finding strategy. It is likely in this scenario that the IMS presentation represents acute HIV sero-conversion; this is supported by the fact that 32.3% of these diagnoses had an HIV-negative test in the 12 months prior to diagnosis. The effect of introducing routine testing in people presenting with IMS has significant potential impact for both individual and public health in terms of avoiding harm (reducing late diagnosis) and minimising the risk of onward transmission (access to Treatment as Prevention). Only half of those testing positive had a low CD4 cell count at diagnosis; in those who had a low count it is likely this was—in a significant number—due to the reversible fall in CD4 cell counts associated with seroconversion. Given all these factors it is especially important that these individuals should be offered HIV testing and strategies and guidelines need to emphasise this.

### Linkage to care

Data on linkage to care and treatment initiation were reported for 58.1% of those testing positive. The high level of missing data is likely to be due to the testing and study reporting taking place in non-HIV settings and the difficulty obtaining clinical data about subsequent medical attendances from HIV services. Whilst we cannot make assumptions about the individuals with missing data, the lack of clinical data accessible to a testing site is of concern as engagement in care is a key element of the effective monitoring of a testing programme.

Where we have data available our rates for linkage to care exceed 90% and occurs in a timely fashion; this is higher than that reported elsewhere [12–14]. If missing data is treated as failure to transfer to care this fall to 54%. It is probable the true proportion lies somewhere between. Of those reported as being promptly linked to care 10.4% were reported to be either LTFU or dead within 12 months of diagnosis. All of those who died had a low CD4 count at diagnosis.

The location of the initial testing is likely to strongly influence the accessibility of subsequent clinical data by the testing service. Physical, organisational and specialty separation may all play a part, alongside the lack of clinical utility to the test originator. The outcome is not known for 42% of those testing positive and suggests services lack clear mechanisms to ensure transfer to care has occurred and is documented, with appropriate interventions if it has not taken place.

The fact that data on linkage to care and treatment initiation were only available for 58% of those testing positive, underlines the importance of improved monitoring of linkage to care when testing is introduced in “non-HIV” settings, be it hospital department or general practitioners as in this study or community-based settings. Testing services need to establish robust mechanisms to confirm timely transfer to care with appropriate interventions when this fails to occur.

### Implementation

The ICs confirmed in the HIDES study should be adopted into European and national HIV testing and IC specialty guidelines. Specific focus should be on specialty guidelines, for example respiratory/pulmonology, haematology and internal medicine, many of which frequently do not include recommendations for HIV testing in ICs [15] despite evidence to support the cost effectiveness of testing in these situations. Given the delivery of these programmes are frequent in departments unused to delivering a safe, effective HIV testing programme implementation tools, including those for training and education, can support its introduction and spread. Such tools have been developed through the EU-co-funded project OptTEST [16] and will be further supported through the EU co-funded INTEGRATE Joint action [17].

IC guided HIV testing strategy should be incorporated into guidelines to supplement the current recommendations which mainly focus on testing specific at-risk groups (PWID, MSM) and in certain settings (e.g. sexual health services). Both these strategies require either self-identification of risk and an individual acting on this to present for testing or being asked about possible risks (or assumptions made); additional resources are often required if services are to ‘reach’ out to many of these groups. Whilst effective to a degree these approaches have to date been insufficient to address the epidemic and additional strategies are required. IC guided HIV testing has a number of advantages to contribute; normalisation of HIV testing, reduced stigmatisation, opportunistic nature with reduced associated costs and being independent of the requirement for individual or health care worker to carry out a risk assessment.

### Limitations

There are a number of potential limitations to our study. As we do not report risk group data and test offer and offer outcome we cannot exclude the potential bias of targeted offer of testing based on belonging to a risk group. Most services had no mechanism and limited capacity to capture test offer and outcome, however other studies have reported high acceptance rate of HIV test offer across a variety of healthcare settings[6]. Furthermore, we are unable to report denominator data for total number of patients attending for management of the specific IC; this was not an objective of this study, but as reported elsewhere [6] coverage is generally low across most ICs, apart from TB and some AIDS defining conditions in some regions. All ICs were not offered uniformly across the region and this again may introduce bias by different services in different regions having variation in the risk profiles of those who attend. Transferability cannot be assumed as many of those involved are already advocates of HIV testing and coverage may be less complete if rolled out to less engaged clinicians. Finally, recall bias may have affected reporting of previous potential HIV related symptoms and illnesses and under-estimated missed opportunities for earlier diagnosis.

## Conclusion

The HIDES II study confirms IC status for two important and common conditions; lymphadenopathy and pneumonia. Within the study HIV testing in 10 conditions demonstrated HIV prevalences exceeding > 0.1%. This was not demonstrated for the remaining four conditions; neuropathy, psoriasis, anal cancer and lung cancer, as relatively low numbers of patients were tested and there were few events; further work is required for these conditions. As infectious mononucleosis-like syndrome can mimic acute HIV sero-conversion illness and has the highest HIV prevalence, this IC in particular affords opportunities for earlier HIV diagnosis and transmission interruption. We would therefore recommend these ICs to be included in European and national HIV testing and IC specialty guidelines. Further work is required to support implementation of IC driven HIV testing as this strategy has the potential to significantly impact on undiagnosed HIV and late diagnosis with well recognised associated individual and public health benefits.

## Supporting information

S1 Appendix List of sites and settings. (DOCX)

S2 Appendix List of EC/IRB per country. (DOCX)

S3 Appendix Data collection form. (PDF)

S1 Table Characteristics of patients testing HIV+, stratified by region. (DOCX)
