# Supplementary material for: Improving the evidence for indicator condition guided HIV testing in Europe: Results from the HIDES II Study – 2012 – 2015
Source: PLoS One. 2019 Aug 13;14(8):e0220108. doi: 10.1371/journal.pone.0220108 (PMC6692030; doi:10.1371/journal.pone.0220108)
Supplement: S1 Appendix — (DOCX) [file pone.0220108.s001.docx]

| **S1 Appendix. List of Sites and Settings** | | | |
| --- | --- | --- | --- |
| **Country**  (new countries in HIDES2 **- in bold**) | **City** | **Site**  (new sites recruited for HIDES2 - **in bold**) | **Setting where patients where seen:**  Hospital Out-patient  Hospital In-patient  Primary Care |
| Austria | Innsbruck | Medical University Innsbruck | Hospital Out-patient  Hospital In-patient  Primary Care |
| Belarus | Minsk | Belarus State Medical University | Hospital Out-patient  Hospital In-patient |
|  | **Gomel** | **Gomel state Medical University** | **Hospital In-patient** |
| Belgium | Brussels | CHU Saint-Pierre | Hospital Out-patient  Hospital In-patient  Primary Care |
| **Bosnia & Herzegovina** | **Sarajevo** | **Klinicki centar Univerziteta Sarajevo (KCUS)** | **Hospital In-patient** |
| Croatia | Zagreb | University Hospital of Infectious Diseases | Hospital Out-patient  Hospital In-patient |
| Denmark | **Odense** | **OUH, Odense Universitetshospital, Forskningsenheden** | **Hospital Out-patient**  **Hospital In-patient** |
|  | **Roskilde** | **Sjællands Universitets Hospital** | **Hospital Out-patient**  **Hospital In-patient** |
| **France** | **Paris** | **Hopital de la Pitié-Salpêtriére** | **Hospital In-patient** |
|  | **Paris** | **Hôspital Bichat - Claude Bernard** | **Hospital Out-patient**  **Hospital In-patient** |
|  | **Saint Denis** | **Hôspital Delafontaine** | **Hospital In-patient** |
|  | **Pontoise** | **Centre hospitalier René Dubos** | **Hospital Out-patient**  **Hospital In-patient** |
|  | **Saint-André** | **Hôspital Saint-André** | **Hospital In-patient** |
|  | **Clermont-Ferrand** | **CHU de Clermont-Ferrand** | **Hospital Out-patient**  **Hospital In-patient** |
|  | **Rennes** | **Hôspital Pontchaillou** | **Hospital In-patient** |
|  | **Tourcoing** | **Centre hospitalier de Tourcoing** | **Hospital In-patient** |
|  | **Fort-de- France, Martinique** | **Centre Hospitalier Universitaire de Fort-de-France** | **Hospital Out-patient**  **Hospital In-patient** |
| **Georgia** | **Tblisi** | **Infectious Dis, AIDS, and Clin. Immunology Centre** | **Hospital Out-patient** |
| Germany | Bonn | University Hospital Bonn | Hospital Out-patient |
|  | **Kiel** | **Universitäts-Hautklinik Kiel** | **Hospital Out-patient** |
| **Greece** | **Athens** | **Ippokration General Hospital** | **Hospital Out-patient**  **Hospital In-patient** |
| **Israel** | **Rehovot** | **AIDS Center (Neve Or)** | **Hospital Out-patient**  **Hospital In-patient** |
| Italy | **Milan** | **San Paolo Hospital** | **Hospital Out-patient**  **Hospital In-patient** |
|  | **Catania** | **U.O. Malattie Infettive Università di Catania** | **Hospital Out-patient**  **Hospital In-patient** |
| Poland | Bialystok | Wojewodzki Szpital Specjalistyczny | Hospital Out-patient  Hospital In-patient |
| **Romania** | **Timişoara** | **University of Medicine and Pharmacy "Victor Babes"** | **Hospital Out-patient**  **Hospital In-patient** |
| **Serbia** | **Belgrade** | **Clinical Center of Serbia** | **Hospital Out-patient**  **Hospital In-patient** |
| Spain | **San Sebastian** | **Hospital Donostia** | **Hospital Out-patient**  **Hospital In-patient** |
|  | **Valencia** | **Consorcio Hosp. General Univ. de Valencia** | **Hospital Out-patient** |
|  | **Vigo** | **Complexo Xeral Cies de Vigo** | **Hospital Out-patient** |
|  | **Elche** | **Hospital Universitario de Elche** | **Hospital Out-patient**  **Hospital In-patient** |
|  | **Barcelona** | **CEEISCAT** | **Primary Health care clinics** |
| **Switzerland** | **St. Gallen** | **Kantonsspital St. Gallen** | **Hospital Out-patient**  **Hospital In-patient** |
| The Netherlands | Amsterdam | Onze Lieve Vrouwe Gasthuis | Hospital Out-patient  Hospital In-patient |
| Ukraine | Kharkiv | Kharkiv State Medical University | Hospital In-patient |
|  | **Luhansk** | **Luhansk AIDS Center** | **Hospital Out-patient**  **Hospital In-patient**  **Primary Healthcare** |
| UK | London | Chelsea and Westminster | Hospital Out-patient  Hospital In-patient |
|  | **London** | **Homerton University Hospital** | **Hospital Out-patient**  **Hospital In-patient** |
|  | **Leicester** | **Leicester Royal Infirmary** | **Hospital Out-patient**  **Hospital In-patient** |
|  | **Leeds** | **St James's University Hospital, Leeds** | **Hospital Out-patient**  **Hospital In-patient** |
|  | **Huddersfield** | **Huddersfield Royal Infirmary** | **Hospital Out-patient** |
|  | **London** | **North End Medical Centre** | **Primary Health Care** |
|  | **Brighton** | **Stanford Medical Centre** | **Primary Health Care** |
|  | **Manchester** | **The Docs Surgery** | **Primary Health Care** |
|  | **London** | **Kings College Health Centre** | **Primary Health Care** |
|  | **London** | **Gladstone Medical Centre** | **Primary Health Care** |
