# Supplementary material for: Improving the evidence for indicator condition guided HIV testing in Europe: Results from the HIDES II Study – 2012 – 2015
Source: PLoS One. 2019 Aug 13;14(8):e0220108. doi: 10.1371/journal.pone.0220108 (PMC6692030; doi:10.1371/journal.pone.0220108)
Supplement: S2 Appendix — (DOCX) [file pone.0220108.s002.docx]

**S2 Appendix. List of EC/IRB per Country**

Ethical approval was obtained where required in the participating countries in line with National requirements. As HIV testing was performed routinely as part of the investigation of the IC, consent was obtained verbally where required. Please find a list below of the Ethical Committees that approved the study

Austria: Ethikkommission, Medizinische Universität Innsbruck. Geschäftsstelle, Innrain 43, A-6020 Innsbruck, Austria

Belarus: Health Care Facility City Clinical Infectious Hospital (EC), 220002, Minsk, ul.Kropotkina 76, Belarus

Belgium: Comite Local D’Ethique Hospitalier, CHU Saint-Pierre, Rue Haute 322 – 1000 Brussels

Bosnia & Herzegovina: Etički Komitet, Klinički centar Univerziteta u Sarajevu. Bolnička 25, 71000 Sarajevo
Bosna i Hercegovina

Croatia: Local Ethics Committee, University Hospital for Infectious Diseases, Mirogojska 8, 10 000 Zagreb, Croatia.

Denmark: Regional Ethics Committee, Region Hovedstaden, Kongens Vænge 2, 3400 Hillerød, Denmark.

France: CPP Ile-de-France VI 47, Groupe Hospitalier Pitié-Salpêtrière. Boulevard de l’Hopital 75013, Paris, France.

Georgia: Institutional Review Board, 16 Al. Kazbegi Ave, Tbilisi 0160, Georgia.

Germany: Ethik-Kommission der Medizinischen Fakultät der Universität Bonn. Biomedizinisches Zentrum, Sigmund-Freud-Str. 25, 53105 Bonn.

Greece: not required

Israel: Ethics Committee. Kaplan Medical Center. 1, Rechovot, 76100 Israel.

Italy: Comitato Etico, Piazza S. Maria di Gesú n.5, 95123 Catania.

Romania: Comisia de Etica. Bd. Iosif Bulbuca 10, 300736 Timisoara, Romania

Serbia: Klinicki Centar Srbije, Eticki Odbor, Pasterova 2, Beograd 11000, Serbia.

Spain: CEIC, Sancho El Sabio, 35, 20010 Donostia-San Sebastián and CEIC, Consorcio Hospital General Universitario de Valencia. Avda. Tres Cruces nº 2 Pabellón B-3, 4ª planta, 46014 – Valencia and CEIC, Hospital Universitari Germans Trias I Pujol. Badalona. Crta. De Canyet, s/n, 08916 Badalona.

Switzerland: not required

The Netherlands: VCMO (Verenigde Commissies Mensgebonden Onderzoek), Secretariaat, Koekoekslaan 1, 3435 CM Nieuwegein, Postbus 2500, 3430 EM Nieuwegen, The Netherlands.

Ukraine: Ethics Committee, Sverdlova street 1, Lysychansk, Luhansk Oblast, 93106, Ukraine and Hospital EC, Heroiv Stalinhradu Ave, 160, Kharkiv, Kharkivs'ka Oblast, 61000, Ukraine.

United Kingdom: not required
