# Supplementary material for: Improving the evidence for indicator condition guided HIV testing in Europe: Results from the HIDES II Study – 2012 – 2015
Source: PLoS One. 2019 Aug 13;14(8):e0220108. doi: 10.1371/journal.pone.0220108 (PMC6692030; doi:10.1371/journal.pone.0220108)
Supplement: S3 Appendix — (PDF) [file pone.0220108.s003.pdf]

HIDES Enrolment

Patient ID:

____
((XXXX-XXXXX))

Section A: Demography

Year of birth:
(MUST be between the age of 18 and 65)

____
((YYYY))

Gender:

- ◯ Male
- ◯ Female

Ethnicity:

- ◯ Caucasian
- ◯ Asian
- ◯ African
- ◯ Unknown

Section B: Indicator Disease

Patient presenting with: (based on treating physician's clinical or microbiological diagnosis)

- ◯ Malignant lymphoma (irrespective of type)
- ◯ Cervical dysplasia/cancer (CIN II and above)
- ◯ Anal dysplasia/cancer
- ◯ Hepatitis B (acute or chronic - and irrespective of time of diagnosis relative to time of survey)
- ◯ Hepatitis C (acute or chronic - and irrespective of time of diagnosis relative to time of survey)
- ◯ Hepatitis B+C (acute or chronic - and irrespective of time of diagnosis relative to time of survey)
- ◯ Ongoing mononucleosis-like illness
- ◯ Unexplained leukocytopenia or thrombocytopenia lasting at least 4 weeks
- ◯ Seborrheic dermatitis/exanthema
- ◯ Pneumonia (admitted to hospital at least 24h)
- ◯ Unexplained lymphadenopathy
- ◯ Peripheral neuropathy of unknown cause (diagnosed by neurologist)
- ◯ Primary lung cancer
- ◯ Severe or recalcitrant psoriasis (newly diagnosed)
- ◯ Uveitis
- ◯ Periodontitis
- ◯ Acute coronary syndrome/myocardial infarction
- ◯ Pulmonary hypertension

((Only one box ticked allowed))

Section C: HIV Test Results

Previous HIV serological status (patients must NOT be known to be HIV infected at the time of survey)

Previously tested for HIV:

- ◯ Yes
- ◯ No
- ◯ Unknown

Most recent previous negative HIV test: ______________________ ((DD-MM-YYYY))

Total number of previous negative HIV tests: ______________________

HIV test result:
◯ Positive
◯ Negative

Date of blood sample: ______________________ ((DD-MM-YYYY))

Patient received test result:
◯ Yes
◯ No
◯ Unknown

Patient successfully transferred to HIV specialist care:
◯ Yes
◯ No
◯ Unknown

Section D: Setting

Setting patient was seen:
◯ Hospital out-patient department/clinic
◯ Hospital in-patient/ward
◯ Primary care

Section E: HIV Infected

CD4 cell counts (closest to diagnosis) value: ______________________

Date: ______________________ ((DD-MM-YYYY))

HIV-RNA values: ______________________ ((Units))

Date: ______________________ ((DD-MM-YYYY))

Section F: Additional Data Items

Sexual orientation:
◯ Heterosexual
◯ Homosexual
◯ Bisexual
◯ Unknown

Active intravenous drug use:
◯ Yes
◯ No
◯ Unknown

Has the patient had any signs of less serious HIV related symptoms within the last 5 years:	☐ Mononucleosis-like illness<br>☐ Oral candidiasis<br>☐ Herpes zoster<br>☐ Unexplained leukocytopenia or thrombocytopenia<br>☐ Seborrheic dermatitis/exanthema<br>☐ None
Diagnosed sexually transmitted diseases within the last 5 years:	☐ Gonorrhoea<br>☐ Syphilis<br>☐ Other ulcerative genital conditions<br>☐ Chlamydia<br>☐ Unspecified<br>☐ None

HBV

Any previous test of HBV:	○ Yes<br>○ No
Test result:	○ Positive<br>○ Negative
Date:	______________________<br>((DD-MM-YYYY))

HCV

Any previous test of HCV:	○ Yes<br>○ No
Test result:	○ Positive<br>○ Negative
Date:	______________________<br>((DD-MM-YYYY))

Hospitalization

Any hospitalization within the last 5 years:	○ Yes<br>○ No

Due to severe opportunistic infections (including AIDS defining):

Diagnose:
- ◯ BCNE
- ◯ CANO
- ◯ CMVR
- ◯ CMVO
- ◯ CRCO
- ◯ CRSP
- ◯ CRVC
- ◯ FBLS
- ◯ HERP
- ◯ HIST
- ◯ ISDI
- ◯ KS
- ◯ LEIS
- ◯ LEU
- ◯ MC
- ◯ MCP
- ◯ MCX
- ◯ MCXO
- ◯ NHLB
- ◯ NHLI
- ◯ NHLU
- ◯ NHLP
- ◯ PCP
- ◯ SAM
- ◯ TOX

Please specify: ______________________________

Date of diagnosis: ______________________________
((DD-MM-YYYY))

Diagnose:
- ◯ BCNE
- ◯ CANO
- ◯ CMVR
- ◯ CMVO
- ◯ CRCO
- ◯ CRSP
- ◯ CRVC
- ◯ FBLS
- ◯ HERP
- ◯ HIST
- ◯ ISDI
- ◯ KS
- ◯ LEIS
- ◯ LEU
- ◯ MC
- ◯ MCP
- ◯ MCX
- ◯ MCXO
- ◯ NHLB
- ◯ NHLI
- ◯ NHLU
- ◯ NHLP
- ◯ PCP
- ◯ SAM
- ◯ TOX

Please specify: ______________________________

Date of diagnosis:

____
((DD-MM-YYYY))

Diagnose:

- ◯ BCNE
- ◯ CANO
- ◯ CMVR
- ◯ CMVO
- ◯ CRCO
- ◯ CRSP
- ◯ CRVC
- ◯ FBLS
- ◯ HERP
- ◯ HIST
- ◯ ISDI
- ◯ KS
- ◯ LEIS
- ◯ LEU
- ◯ MC
- ◯ MCP
- ◯ MCX
- ◯ MCXO
- ◯ NHLB
- ◯ NHLI
- ◯ NHLU
- ◯ NHLP
- ◯ PCP
- ◯ SAM
- ◯ TOX

Please specify:

____

Date of diagnosis:

____
((DD-MM-YYYY))

Diagnose:

- ◯ BCNE
- ◯ CANO
- ◯ CMVR
- ◯ CMVO
- ◯ CRCO
- ◯ CRSP
- ◯ CRVC
- ◯ FBLS
- ◯ HERP
- ◯ HIST
- ◯ ISDI
- ◯ KS
- ◯ LEIS
- ◯ LEU
- ◯ MC
- ◯ MCP
- ◯ MCX
- ◯ MCXO
- ◯ NHLB
- ◯ NHLI
- ◯ NHLU
- ◯ NHLP
- ◯ PCP
- ◯ SAM
- ◯ TOX

Please specify: ______________________________

Date of diagnosis: ______________________________
((DD-MM-YYYY))

Diagnose:

- ◯ BCNE
- ◯ CANO
- ◯ CMVR
- ◯ CMVO
- ◯ CRCO
- ◯ CRSP
- ◯ CRVC
- ◯ FBLS
- ◯ HERP
- ◯ HIST
- ◯ ISDI
- ◯ KS
- ◯ LEIS
- ◯ LEU
- ◯ MC
- ◯ MCP
- ◯ MCX
- ◯ MCXO
- ◯ NHLB
- ◯ NHLI
- ◯ NHLU
- ◯ NHLP
- ◯ PCP
- ◯ SAM
- ◯ TOX

Please specify: ______________________________

Date of diagnosis:

____
((DD-MM-YYYY))

Diagnose:

- ◯ BCNE
- ◯ CANO
- ◯ CMVR
- ◯ CMVO
- ◯ CRCO
- ◯ CRSP
- ◯ CRVC
- ◯ FBLS
- ◯ HERP
- ◯ HIST
- ◯ ISDI
- ◯ KS
- ◯ LEIS
- ◯ LEU
- ◯ MC
- ◯ MCP
- ◯ MCX
- ◯ MCXO
- ◯ NHLB
- ◯ NHLI
- ◯ NHLU
- ◯ NHLP
- ◯ PCP
- ◯ SAM
- ◯ TOX

Please specify:

____

Date of diagnosis:

____
((DD-MM-YYYY))

Diagnose:

◯ BCNE
◯ CANO
◯ CMVR
◯ CMVO
◯ CRCO
◯ CRSP
◯ CRVC
◯ FBLS
◯ HERP
◯ HIST
◯ ISDI
◯ KS
◯ LEIS
◯ LEU
◯ MC
◯ MCP
◯ MCX
◯ MCXO
◯ NHLB
◯ NHLI
◯ NHLU
◯ NHLP
◯ PCP
◯ SAM
◯ TOX

Please specify:

____

Date of diagnosis:

____
((DD-MM-YYYY))

Diagnose:

◯ BCNE
◯ CANO
◯ CMVR
◯ CMVO
◯ CRCO
◯ CRSP
◯ CRVC
◯ FBLS
◯ HERP
◯ HIST
◯ ISDI
◯ KS
◯ LEIS
◯ LEU
◯ MC
◯ MCP
◯ MCX
◯ MCXO
◯ NHLB
◯ NHLI
◯ NHLU
◯ NHLP
◯ PCP
◯ SAM
◯ TOX

Please specify:

____

Date of diagnosis: ______________________ ((DD-MM-YYYY))

Other severe infections/cancers:

Diagnose:

- ◯ ALL
- ◯ AML
- ◯ ANUS
- ◯ BACT
- ◯ BLAD
- ◯ BRCA
- ◯ CERV
- ◯ CLL
- ◯ CML
- ◯ COLO
- ◯ COTC
- ◯ ENDO
- ◯ HDL
- ◯ KIDN
- ◯ LIVR
- ◯ LUNG
- ◯ LIPC
- ◯ MALM
- ◯ MEAC
- ◯ MENI
- ◯ MEOC
- ◯ MESC
- ◯ MULM
- ◯ PENC
- ◯ PERI
- ◯ PNEU
- ◯ PROS
- ◯ PYEL
- ◯ OSTI
- ◯ RECT
- ◯ STOM
- ◯ TESE
- ◯ UTER

Date of diagnosis: ______________________ ((DD-MM-YYYY))

Diagnose:

- ◯ ALL
- ◯ AML
- ◯ ANUS
- ◯ BACT
- ◯ BLAD
- ◯ BRCA
- ◯ CERV
- ◯ CLL
- ◯ CML
- ◯ COLO
- ◯ COTC
- ◯ ENDO
- ◯ HDL
- ◯ KIDN
- ◯ LIVR
- ◯ LUNG
- ◯ LIPC
- ◯ MALM
- ◯ MEAC
- ◯ MENI
- ◯ MEOC
- ◯ MESC
- ◯ MULM
- ◯ PENC
- ◯ PERI
- ◯ PNEU
- ◯ PROS
- ◯ PYEL
- ◯ OSTI
- ◯ RECT
- ◯ STOM
- ◯ TESE
- ◯ UTER

Date of diagnosis:

____
((DD-MM-YYYY))

Diagnose:	◯ ALL<br>◯ AML<br>◯ ANUS<br>◯ BACT<br>◯ BLAD<br>◯ BRCA<br>◯ CERV<br>◯ CLL<br>◯ CML<br>◯ COLO<br>◯ COTC<br>◯ ENDO<br>◯ HDL<br>◯ KIDN<br>◯ LIVR<br>◯ LUNG<br>◯ LIPC<br>◯ MALM<br>◯ MEAC<br>◯ MENI<br>◯ MEOC<br>◯ MESC<br>◯ MULM<br>◯ PENC<br>◯ PERI<br>◯ PNEU<br>◯ PROS<br>◯ PYEL<br>◯ OSTI<br>◯ RECT<br>◯ STOM<br>◯ TESE<br>◯ UTER
Date of diagnosis:	____________________________<br>((DD-MM-YYYY))

Diagnose:

- ◯ ALL
- ◯ AML
- ◯ ANUS
- ◯ BACT
- ◯ BLAD
- ◯ BRCA
- ◯ CERV
- ◯ CLL
- ◯ CML
- ◯ COLO
- ◯ COTC
- ◯ ENDO
- ◯ HDL
- ◯ KIDN
- ◯ LIVR
- ◯ LUNG
- ◯ LIPC
- ◯ MALM
- ◯ MEAC
- ◯ MENI
- ◯ MEOC
- ◯ MESC
- ◯ MULM
- ◯ PENC
- ◯ PERI
- ◯ PNEU
- ◯ PROS
- ◯ PYEL
- ◯ OSTI
- ◯ RECT
- ◯ STOM
- ◯ TESE
- ◯ UTER

Date of diagnosis:

____
((DD-MM-YYYY))

Diagnose:

- ◯ ALL
- ◯ AML
- ◯ ANUS
- ◯ BACT
- ◯ BLAD
- ◯ BRCA
- ◯ CERV
- ◯ CLL
- ◯ CML
- ◯ COLO
- ◯ COTC
- ◯ ENDO
- ◯ HDL
- ◯ KIDN
- ◯ LIVR
- ◯ LUNG
- ◯ LIPC
- ◯ MALM
- ◯ MEAC
- ◯ MENI
- ◯ MEOC
- ◯ MESC
- ◯ MULM
- ◯ PENC
- ◯ PERI
- ◯ PNEU
- ◯ PROS
- ◯ PYEL
- ◯ OSTI
- ◯ RECT
- ◯ STOM
- ◯ TESE
- ◯ UTER

Date of diagnosis:

____
((DD-MM-YYYY))

Diagnose:

- ◯ ALL
- ◯ AML
- ◯ ANUS
- ◯ BACT
- ◯ BLAD
- ◯ BRCA
- ◯ CERV
- ◯ CLL
- ◯ CML
- ◯ COLO
- ◯ COTC
- ◯ ENDO
- ◯ HDL
- ◯ KIDN
- ◯ LIVR
- ◯ LUNG
- ◯ LIPC
- ◯ MALM
- ◯ MEAC
- ◯ MENI
- ◯ MEOC
- ◯ MESC
- ◯ MULM
- ◯ PENC
- ◯ PERI
- ◯ PNEU
- ◯ PROS
- ◯ PYEL
- ◯ OSTI
- ◯ RECT
- ◯ STOM
- ◯ TESE
- ◯ UTER

Date of diagnosis:

____
((DD-MM-YYYY))

Diagnose:	◯ ALL<br>◯ AML<br>◯ ANUS<br>◯ BACT<br>◯ BLAD<br>◯ BRCA<br>◯ CERV<br>◯ CLL<br>◯ CML<br>◯ COLO<br>◯ COTC<br>◯ ENDO<br>◯ HDL<br>◯ KIDN<br>◯ LIVR<br>◯ LUNG<br>◯ LIPC<br>◯ MALM<br>◯ MEAC<br>◯ MENI<br>◯ MEOC<br>◯ MESC<br>◯ MULM<br>◯ PENC<br>◯ PERI<br>◯ PNEU<br>◯ PROS<br>◯ PYEL<br>◯ OSTI<br>◯ RECT<br>◯ STOM<br>◯ TESE<br>◯ UTER
Date of diagnosis:	______________________________<br>((DD-MM-YYYY))

Diagnose:

◯ ALL
◯ AML
◯ ANUS
◯ BACT
◯ BLAD
◯ BRCA
◯ CERV
◯ CLL
◯ CML
◯ COLO
◯ COTC
◯ ENDO
◯ HDL
◯ KIDN
◯ LIVR
◯ LUNG
◯ LIPC
◯ MALM
◯ MEAC
◯ MENI
◯ MEOC
◯ MESC
◯ MULM
◯ PENC
◯ PERI
◯ PNEU
◯ PROS
◯ PYEL
◯ OSTI
◯ RECT
◯ STOM
◯ TESE
◯ UTER

Date of diagnosis:

____
((DD-MM-YYYY))

Completed by and date

Completed by:

____
((investigator's initials))

Date completed:

____
((DD-MM-YYYY))
