# Supplementary material for: Improving the evidence for indicator condition guided HIV testing in Europe: Results from the HIDES II Study – 2012 – 2015
Source: PLoS One. 2019 Aug 13;14(8):e0220108. doi: 10.1371/journal.pone.0220108 (PMC6692030; doi:10.1371/journal.pone.0220108)
Supplement: S1 Table — (DOCX) [file pone.0220108.s004.docx]

S1 Table. Characteristics of patients testing HIV+, stratified by Region

|  |  | All | | South | | Central | | North | | East | | P |
| --- | --- | --- | --- | --- | --- | --- | --- | --- | --- | --- | --- | --- |
|  |  | N | % | N | % | N | % | N | % | N | % |  |
| All |  | 303 | 100 | 27 | 8.9 | 12 | 4.0 | 41 | 13.5 | 223 | 73.6 |  |
| Sexual | Heterosexual | 203 | 67.0 | 11 | 40.7 | 3 | 25.0 | 8 | 19.5 | 181 | 81.2 | <0.0001 |
| Orientation | Homosexual | 57 | 18.8 | 11 | 40.7 | 7 | 58.3 | 14 | 34.1 | 25 | 11.2 |  |
|  | Bisexual. | 5 | 1.7 | 1 | 3.7 | 1 | 8.3 | 0 | 0.0 | 3 | 1.3 |  |
|  | Unknown | 38 | 12.5 | 4 | 14.8 | 1 | 8.3 | 19 | 46.3 | 14 | 6.3 |  |
| Active | Yes | 63 | 20.8 | 1 | 3.7 | 1 | 8.3 | 2 | 4.9 | 59 | 26.5 | <0.0001 |
|  | No | 193 | 63.7 | 23 | 85.2 | 11 | 91.7 | 23 | 56.1 | 136 | 61.0 |  |
|  | Unknown | 47 | 15.5 | 3 | 11.1 | 0 | 0.0 | 16 | 39.0 | 28 | 12.6 |  |
| Prior Hepatitis | No | 197 | 65.0 | 23 | 85.2 | 8 | 66.7 | 13 | 31.7 | 153 | 68.6 | <0.0001 |
| B test | Yes | 82 | 27.1 | 4 | 14.8 | 4 | 33.3 | 13 | 31.7 | 61 | 27.4 |  |
|  | Unknown | 24 | 7.9 | 0 | 0.0 | 0 | 0.0 | 15 | 36.6 | 9 | 4.0 |  |
| Prior Hepatitis | No | 182 | 60.1 | 23 | 85.2 | 7 | 58.3 | 14 | 34.1 | 138 | 61.9 | <0.0001 |
| C test | Yes | 100 | 33.0 | 4 | 14.8 | 4 | 33.3 | 12 | 29.3 | 80 | 35.9 |  |
|  | Unknown | 21 | 6.9 | 0 | 0.0 | 1 | 8.3 | 15 | 36.6 | 5 | 2.2 |  |
| Prior | No | 228 | 75.2 | 19 | 70.4 | 11 | 91.7 | 21 | 51.2 | 177 | 79.4 | <0.0001 |
| Hospitalisation | Yes | 53 | 17.5 | 8 | 29.6 | 1 | 8.3 | 5 | 12.2 | 39 | 17.5 |  |
|  | Unknown | 22 | 7.3 | 0 | 0.0 | 0 | 0.0 | 15 | 36.6 | 7 | 3.1 |  |
| Late presenter^1^ | CD4<350 | 178 | 66.4 | 19 | 70.4 | 6 | 50.0 | 16 | 45.7 | 137 | 70.6 | 0.019 |
| High VL^2^ | >100,000 | 220 | 83.0 | 21 | 80.8 | 11 | 91.7 | 26 | 76.5 | 162 | 83.9 | 0.60 |
|  |  | Median | IQR | Median | IQR | Median | IQR | Median | IQR | Median | IQR |  |
| CD4^1^ | /mm^3^ | 230 | 95 – 430 | 98 | 42 – 406 | 348 | 70 – 423 | 386 | 125 – 524 | 227 | 102 – 390 | 0.15 |
| VL^2^ | Log_10_ | 5.2 | 4.6 – 5.8 | 5.4 | 4.9 – 5.7 | 4.9 | 4.5 – 5.4 | 5.4 | 4.5 – 6.0 | 5.1 | 4.6 – 5.8 | 0.22 |

IQR; interquartile range. P-value from chi-squared test for categorical variables and Wilcoxon test for continuous variables. IDU; intravenous drug user. VL; viral HIV load ^1^CD4 count was available for N=268 persons; 27 from South, 12 from Central, 35 from North and 194 from East (p=0.11 comparing proportion with missing data across regions). Late presentation was defined as a CD4 count < 350/mm^3^. ^2^HIV-RNA data were available for N=266 persons; 26 from South, 12 from Central, 41 from North and 194 from East (p=0.21 comparing proportion with missing data across regions). High viral load was defined as a viral load > 5 log_10_ copies/ml
